# A scoping review on the decision-making dynamics for accepting or refusing the COVID-19 vaccination among adolescent and youth populations

**DOI:** 10.1186/s12889-023-15717-5

**Published:** 2023-04-28

**Authors:** Roger Blahut, Amanda Flint, Elaina Orlando, Joelle DesChatelets, Asif Khowaja

**Affiliations:** 1 Faculty of Applied Health Sciences, Brock University, 1812 Sir Isaac Brock Way, ON L2S 3A1 St Catharines, Canada; 2 Niagara Health System, St Catharines, ON Canada

**Keywords:** Acceptance, Hesitancy, Youth, Adolescent, COVID-19 vaccine

## Abstract

**Background:**

Global COVID-19 vaccinations rates among youth and adolescent populations prove that there is an opportunity to influence the acceptance for those who are unvaccinated and who are hesitant to receive additional doses. This study aimed to discover the acceptance and hesitancy reasons for choosing or refusing to be vaccinated against COVID-19.

**Methods:**

A scoping review was conducted, and articles from three online databases, PubMed, Wiley, and Cochrane Library, were extracted and screened based on exclusion and PICOs criteria. A total of 21 studies were included in this review. Data highlighting study attributes, characteristics, and decision-making dynamics were extracted from the 21 studies and put into table format.

**Results:**

The results showed that the primary drivers for accepting the COVID-19 vaccine include protecting oneself and close family/friends, fear of infection, professional recommendations, and employer obligations. Primary hesitancy factors include concerns about safety and side effects, effectiveness and efficacy, lack of trust in pharmaceuticals and government, conspiracies, and perceiving natural immunity as an alternative.

**Conclusions:**

This scoping review recommends that further research should be conducted with adolescent and youth populations that focus on identifying health behaviors and how they relate to vaccine policies and programs.

**Supplementary Information:**

The online version contains supplementary material available at 10.1186/s12889-023-15717-5.

## Background

The Coronavirus Disease-2019 (COVID-19) pandemic has affected the globe on a tremendous scale. As of March 2023, around 6.8 million deaths are a result of COVID-19 on a worldwide scale [1]. As part of efforts to combat COVID-19, vaccinations have been developed and implemented rapidly since late 2020. As of March 2023, approximately 13 million doses of the vaccine have been administered [1]. On a global scale, 69.7% of the total population has received at least one dose [1]. Based on Statistics Canada definition and parameters, the youth population consists of individuals between 15 and 29 [2]. As such, when stratifying by age, youth populations are among the lowest age ranges for having at least one dose of the vaccine [1]. In terms of boosters, this age range also shows lower rates of administration when compared to other age populations globally [1]. There is still room for improvement within these young age brackets that have not been fully vaccinated or received a booster. More efforts are needed to maximize the vaccine coverage and make targeted efforts for universal access to the COVID-19 vaccine.

Research shows vaccine hesitancy has been an issue before COVID-19 [3]. Determinants of vaccine hesitancy have been documented and correlated with factors such as education status, income, and socioeconomic standards [4]. Additionally, studies have examined psychological attributes such as attitude, complacency, constraint, and collective responsibility when measuring the willingness to get vaccinated [5].

It is also important to note that many drivers of vaccine acceptance or hesitancy can be based on health behaviours and theoretical models. Many successful public health programs are predicated on understanding health behaviours and in what context they apply [6]. One such framework is the Health Belief Model (HBM), which applies a theory to change health behaviours based on constructs of risk susceptibility, risk severity, benefits, barriers, self-efficacy, and cues to action [6]. Another model is the Social Cognitive Theory (SCT), which influences health behaviours based on individual experiences, environmental factors, and outside influences [6]. There is a need for further research to understand the reasons for choosing or not choosing to get vaccinated based on these social models [7]. Since the youth and adolescent global population have lower rates of vaccine and booster coverage, there is a need for current and future policymakers to understand these factors and use them as a framework for policy development that considers these health behaviour models in a social, physical, and economical capacity for adolescent and youth populations. Therefore, this scoping review addresses a gap in the literature by synthesizing the current state of knowledge on the factors influencing youth and adolescent decision-making to accept or refuse the COVID-19 vaccine. We examine the primary drivers of accepting or refusing the vaccine and if the decision was based on individual choice or outside influence (e.g. family member, friend, relative, employer, school, health professional).

The primary objective of this study is to collate evidence from online databases available on the acceptance or refusal of the COVID-19 vaccine among the youth and adolescent populations, and map the knowledge gaps and factors influencing their decision-making regarding the COVID-19 vaccine.

## Methods

### Registration and format

This scoping review was conducted according to the Preferred Reporting Items for Systematic Reviews and Meta-Analyses extension for Scoping Reviews (PRISMA-ScR) Statement [8]. An OSF pre-registration was also done based on this scoping review project and the protocol registration is available (https://doi.org/10.17605/OSF.IO/NJZUA).

### Database searches

This review was performed using three online databases: PubMed, Wiley, and Cochrane Library. Databases were searched using key terms related to the factors that affect adolescent and youth decision-making towards COVID-19 vaccine acceptances or refusals from 1 January 2020 to May 2022, in English. Each key word was included as a combination for database searches. Search strategies (see Additional file 1) and keywords include (i) Youth; (ii) Adolescent; (iii) COVID-19 vaccine; (iv) Acceptance; and (v) Refusal.

### Eligibility criteria

Studies were considered eligible if, (i) the target population included youth (15–29 years) and adults (29–64 years) [2]; (ii) the focus of study was related to COVID-19 vaccine acceptance or refusal; (iii) it is written in English; (iv) the paper is an original study; and (v) the timeframe lies between January 1, 2020 to May 1, 2022.

In the case of discordant observations, both screeners reviewed the articles in question and made any judgements for including or excluding the articles. If unresolved, the matter would be discussed with principal co-investigator.

### Title and abstract screening process

One student research assistant (RB) imported retrieved articles into Zotero (reference management software) to remove the duplicates and for citation purposes. An Excel tool was adapted from Lajeunesse, 2021 to guide title and abstract screening [9]. Two student research assistants (RB, AF) screened the title and abstract according to the eligibility criteria based on PICOs (Participants, Interventions, Comparators, Outcomes, Study Design). Each screener assessed half of the articles retrieved (Screener 1: articles 1 – 519, Screener 2: articles 520–1039).

### Full-text study selection

After the initial screening, the full-text documents of chosen articles were downloaded and both student research assistants read the full-text articles for in-depth screening. This in-depth screening process followed similar methods of PICOs from the initial title and abstract screening and also included the eligibility criteria from above. Finally, the data of the eligible articles were extracted, and the two research assistants mapped the knowledge gaps and synthesized the literature into emerging themes and sub-themes. Synthesizing the literature mainly included providing study characteristics and traits, followed by extracting any findings from the study that included results on hesitancy factors/reasons and acceptance factors/reasons for COVID-19 vaccinations.

### Data presentation

Data relevant was extracted using Microsoft Excel and Word software applications. The collection and summary of these study characteristics and themes are presented in table format in the supplementary files and results. Additionally, the graph figures in the results section illustrate the main decision-making factors and their associated frequencies found among the extracted articles.

## Results

### Study screening process

**Fig. 1:**
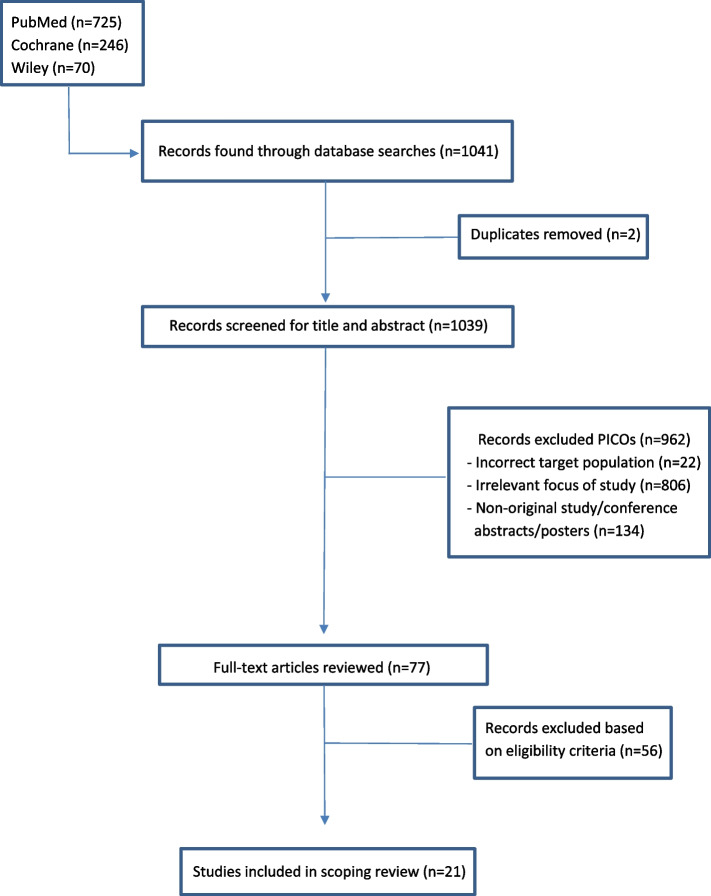
Screening flowchart

As shown in Fig. 1, 21 articles were included in this scoping review following the application of detailed PICOs screening and exclusion criteria.

### Study features

The articles included in the review described study methods sufficiently to extract key information for this review. Study characteristics are highlighted (see Additional file 2). The majority of studies (*n* = 16, 76%) conducted were online cross-sectional surveys or questionnaires. Notable exceptions included two studies with a hybrid of online surveys, telephone interviews, and face-to-face interviews [10, 11]. Another exception is a study that utilized open-ended, text messaging questionnaires to gain qualitative responses [12]. One study conducted focus groups in a qualitative design [13]. Lastly, one study used a discrete choice experiment [14]. Almost all studies (*n* = 19, 90%) obtained their sample population from one country, except two studies that conducted a global survey based on 17 countries and the other based on Canada and France [15, 16]. The applicable country of origin for the study population is indicated as well (see Additional file 2).

Most studies conducted their research during a period after the COVID-19 vaccine was officially announced and ready to be distributed. Six studies were conducted before vaccine rollout but were close to official dates of distribution [12, 15, 17–20]. One study was done during summer 2020, prior to the availability of vaccines [21]. Recruitment methods of most studies (n = 15, 71%) included utilizing social media (e.g.Facebook, Whatsapp) and mass email distribution. Other notable recruitment methods included research and employment services, websites, news portals, and blogs [16, 20, 22, 23]. Convenience sampling was used in eleven studies [10, 11, 15, 16, 18–20, 23–26], non-probability sampling was used by two studies [21, 26] whereas probability-based sampling [22, 27], snowball sampling [28], all-purpose sampling [17], stratified random sampling [21], and weight samples [12] were each used in one study. The remaining studies (*n* = 2, 9%) did not describe sampling technique.

### Study themes

Themes, factors, and influences on vaccination status and intent are indicated (see Additional file 3). Many studies correlated sociodemographic factors with vaccination acceptance or hesitancy. Respondents from studies also indicated personal motivators and decision-making factors related to their acceptance of or hesitancy toward COVID-19 vaccines.

### Socio-demographics related to vaccination status

Overall, older populations were more willing to accept the COVID-19 vaccine than younger populations [10, 11, 21–23, 25]. However, there are some exceptions in which the younger population was more willing to accept the vaccine [16, 18]. The reasoning for this is possibly due to country demographics having a higher distribution of young people (as compared to older adults) and older adults were less likely to use online surveys [18]. Those of higher socioeconomic status and income were more likely to accept the COVID-19 vaccine [10, 15, 23, 28]. Interestingly, one study conducted in Jordan indicated that those who were unemployed were more likely to accept the vaccine than those who were employed [18]. Finally, many studies described that higher education status correlated with increased vaccination acceptance [11, 15, 16, 21–23, 28].

### Decision-making factors and motives for acceptance

**Fig. 2:**
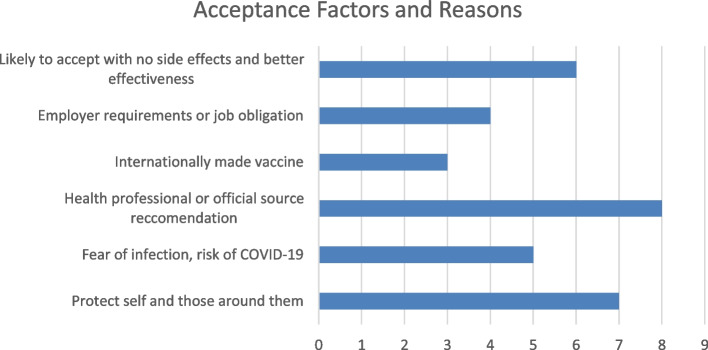
Factors and reasons related to COVID-19 vaccine acceptance

Based on the studies analyzed, the key themes and motivators that relate to COVID-19 vaccination acceptance are presented (see Additional file 3). This showcases aspects of acceptance concerning COVID-19 vaccinations. Figure 2 quantifies and illustrates the main acceptance themes and drivers of the COVID-19 vaccine.

Many respondents from these studies indicated that the main reason for getting vaccinated was to protect themselves from COVID-19 and to protect those around them, such as family and friends [10–12, 15, 23, 27, 29]. Another motivator reported within many of these studies was fear of infection, or perceived risk and seriousness of the virus [17, 20–22, 28]. Participants reported being more likely to accept the vaccine if there was evidence that the vaccine had minimal to no side effects, and that it was effective against COVID-19 [12, 16, 20, 21, 23, 26]. Study participants stated that having a healthcare professional recommend the COVID-19 vaccine or utilizing professional and governmental sources of vaccine information propagates further acceptance [10, 12, 13, 19, 20, 22, 26, 30]. Interestingly, some studies indicated greater likelihood of acceptance for a COVID-19 vaccine that was internationally made [19, 21, 26]. In contrast, one study found that respondents preferred if the vaccine was manufactured within their own country [11]. Two studies also specifically stated that respondents were more accepting of the Pfizer vaccine than other manufacturers [11, 25]. A few studies indicated that employer requirements or the obligation and feeling of wanting to preserve jobs were motivators and reasons for getting vaccinated [23, 26, 27, 29]. Finally, other factors influencing acceptance of the COVID-19 vaccine included: benefits outweighing the risks [10], a desire for more clinical trials to prove efficacy [23, 26], more willingness to vaccinate at vaccine centers of general practitioner’s office [14], a longer vaccine threshold of effectiveness [16], and to avoid travel bans [23].

### Decision-making factors and motives for hesitancy

**Fig. 3:**
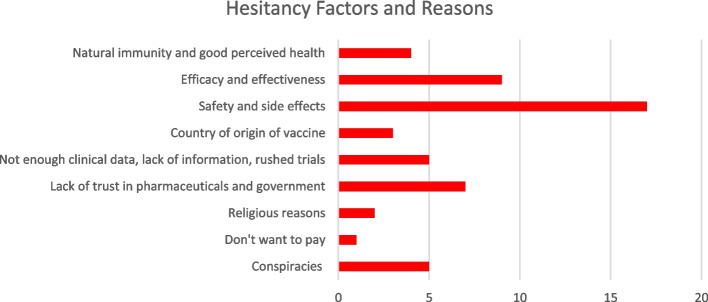
Factors and reasons related to COVID-19 vaccine hesitancy

The key themes relating to COVID-19 vaccine hesitancy are present (see Additional file 3). Figure 3 quantifies and illustrates the main hesitancy themes and drivers of the COVID-19 vaccine.

There was a pervasive theme among most studies that respondents were hesitant to be vaccinated against COVID-19 due to a belief that the vaccines were unsafe, non-efficacious, and had concerning side effects [10–13, 15–21, 23, 25, 26, 28–30]. In combination with this, several studies indicated that respondents felt that COVID-19 vaccine clinical trials were rushed or there was not enough clinical data and information about the vaccine [10, 12, 17, 19, 23, 29]. Several studies described a lack of trust in the pharmaceutical industry and government as deterring respondents from getting the vaccine [10–13, 17, 23, 25]. Similarly, a few studies mentioned that conspiracies against COVID-19, the government, and global plots have resulted in the refusal of the vaccine [12, 18, 20, 24, 30].

In relation to the country where the vaccine was manufactured, two studies found that respondents were hesitant to accept a vaccine if it was made in China [21] or India [19]. Refusing the vaccine due to religious reasons was also a factor in vaccine hesitancy [23, 11]. Some respondents shared that obtaining natural immunity was preferred over getting vaccinated [11, 25, 29]. Those more prone to using social media as information sources were more likely to be hesitant towards vaccination [13, 22, 30]. Other reasons reported for hesitancy about COVID-19 vaccination included: not willing to pay [18], perceived oneself in good health [25, 26], the number of doses needed was too many [16], lower perceived severity of COVID-19 [21, 23], allowing high-risk people to receive the vaccine first [17, 28], lack of support for QR code utilization, which includes technological faults or inabilities to use QR code technologies[14], and lack of information regarding the vaccine [13, 25, 28].

## Discussion

This scoping review highlighted factors contributing to the acceptance or hesitancy towards the COVID-19 vaccination among adolescent and youth populations. Although some socio- demographics were correlated with vaccine decision-making, many of the factors influencing decisions to get vaccinated are based on personal characteristics and outside influences. The Health Behaviour Model (HBM) enforces the theory to change health behaviours based on factors regarding risk susceptibility, risk severity, benefits, and barriers [6]. Social Cognitive Theory (SCT) influences health behaviours based on individual experiences, environmental factors, and outside influences [6]. The findings from this study can be aligned with principles from these models and can further be applied to COVID-19 vaccine policies and practice changes. Furthermore, future interventions and policies regarding COVID-19 vaccinations (or other vaccines) can apply fundamentals from these models and see how young populations’ acceptance and refusal factors relate to these same theories [31].

### Acceptance factors to the HBM

In terms of accepting the vaccine, the main driver for getting the COVID-19 vaccination included protecting oneself and those around them, such as family and friends. This may also tie into the fact that many people were also willing to get the vaccine because of fear of infection or severity of COVID-19. The HBM coincides with this type of behaviour, in which people perceive this as a real threat and show vulnerability. As such, more people are willing to get vaccinated if they perceive this as a threat to their health and loved ones [31]. Other supporting motivators and reasons for willingness to vaccinate include professional recommendations from healthcare providers and other government or official sources. Indeed, trust in the healthcare system and general trust with healthcare providers can improve willingness to vaccinate against COVID-19 [32]. This can play a critical role in public health, healthcare, and government communication where professionalism, honesty, and trust can help to improve vaccination rates [32]. Communication and transparency are vital in regards to HBM, since many respondents claimed that they would be more willing to take the vaccine if the evidence supported that the vaccine caused minimal or no side effects and was also proven to be effective. Other decision-making factors include where the vaccine was manufactured and by whom. These studies showcased that Pfizer and internationally made vaccines – mainly within the European Union or the United States – were generally more trustworthy [19, 21, 26]. Perhaps this larger trust in pharmaceutical industries within these developed nations can be a reason for people to feel more secure and comfortable with taking these international vaccines [33].

### Questionable SCT influencers

An important reason for vaccine acceptance found within this scoping review is the employment aspect. This motivator displayed that some people were willing to vaccinate if their employer expressed willingness or forced mandates. Additionally, some respondents indicated that they felt they had to vaccinate to keep their jobs or preserve them. This is interesting because although this can be classified as an acceptance motivator, it is not necessarily done by personal choice. It is more of an outside influence where people may feel they do not necessarily have a choice in whether to be vaccinated or not. Rather than following the HBM, this type of health behaviour coincides more with SCT, where personal factors and environmental influences correlate with health behaviour [6]. Unfortunately, this may go against the notion of healthy behaviour where economic necessity outweighs personal health, susceptibility, and prevention. The ethical dilemmas of being mandated to vaccinate are concerning, especially among healthcare workers [34]. Within Canada, many people have been put on unpaid leave or termination without compensation, further causing confusion and complex legality issues [35]. With Quebec initiating a tax for those that are unvaccinated, the ethical and economic considerations are even more daunting [36]. Whether this is due to firing unvaccinated workers or proposing vaccine mandates; and thereby limiting the application pool, the economic burdens of workforce shortage has been apparent [37]. All these factors are more concerning given that adolescent and youth populations can be more “forced” into these types of decisions solely based on their perceived economic prosperity and future goals.

### Hesitancy factors to the HBM

On the opposite spectrum, hesitancy factors were highly correlated to COVID-19 vaccine safety, effectiveness, and possible side effects. In relation to this, other reasons such as rushed clinical trials and insufficient clinical data were applicable for many hesitant respondents. In another scoping review, researchers also found that parents were mainly concerned with vaccine efficacy and safety [38]. Although data has been provided and clinical trials revealed vaccine effectiveness, people are still hesitant. This may relate to inconsistent data or poorly communicated evidence from healthcare professionals and government authorities [38]. This distrust is critical to overcome since HBMs coincide with the notion of ensuring these vaccines are safe and effective in dealing with the negative consequences of COVID-19. This also ties into factors for those who rely on social media are more prone to being hesitant, as indicated from respondents in this scoping review. Indeed, misinformation is amplified through social media platforms and is becoming an issue for global public health, especially among younger populations [39]. In relation to younger populations, many respondents specified that they prefer natural immunity and believe they are young and healthy, so they do not need the vaccine. Although some sources state that natural immunity has its limits [40], other research also indicates that natural immunity should not be shunned or ignored as means of policy mandates [41].

Another hesitant motivator is the number of doses. Respondents stated that a higher number of doses were associated with an unwillingness to vaccinate. This can also relate to additional booster shoots as people may be unwilling to get more vaccine jabs. In conjunction with this theme, vaccine effectiveness is also perceived to be lower. As such, people were only more willing to get vaccinated if it was shown that the vaccine has a long duration of effectiveness [16]. All these drivers can relate to the HBM, where these hesitant respondents do not feel as if the vaccine is effective and safe enough to be used for the prevention and severity of COVID-19 [6].. For vaccine manufacturers, this must include transparent information and testing, that is clear and open to all who wish to understand vaccine effectiveness. Additionally, public health organizations and government policymakers need to understand that these health behaviours can be better applied if proper and timely information, evidence, and reputation relate to the perceived health model for these young populations.

Similar to economic reasoning, a hesitant motivator was related to paying for the vaccine [18]. One study showed that respondents were more hesitant in getting the COVID-19 vaccine if they needed to pay for it [18]. Although many countries have utilized taxpayer dollars to distribute the vaccine for free, some respondents were from countries where out-of-pocket expenses were needed to pay for the vaccine. In addition, it should also be noted that some people may have to pay indirect costs associated with getting the vaccine. For example, taking time off or work or paying for travelling costs to get the vaccine may hinder some people from acceptance. Although some countries, such as Canada, help to mitigate these complications by allowing half a day of paid time off, this is mainly accounting for core public administration employees and not the private sector [42]. Other pay of leave arrangements may not work in favour of the individual, and it is entirely dependent on their circumstances and workplace. Possible solutions can be to ensure paid time off for vaccination inoculations or a government allowance fee to propose getting vaccinated for those countries that need to pay for COVID-19 vaccination. Regardless of solutions for tackling this issue, it is imperative that these policy makers realize the willingness of people who will pay for health interventions as it relates to their personal factors and health behaviours. If some cost may be necessary, public health officials must showcase how this initial investment will benefit individuals in the long run based on the HBM of prevention and mitigation of the severity and susceptibility of COVID-19 [6].

### Focusing on young population demographics

An important topic of discussion within this scoping review is understanding the primary drivers of decision-making dynamics within the adolescent and youth population for COVID-19 vaccination status.. This is important as these populations begin to explore new careers and education to help further their own goals and aspirations. This is vital as it can help us investigate whether vaccine status is predicated on whether these populations perceive job, education attainment, and economic gains through receiving the COVID-19 vaccine or not. Understanding how certain policies or decisions on vaccine mandates and status can help to provide better information for these adolescent and youth citizens. This will help to engage in feedback and understand what motives apply to becoming more accepting of COVID-19 vaccinations, especially as booster rates among these populations are low [1]. In addition, utilizing health behaviour models can ensure a framework for how these young populations differ in COVID-19 vaccine uptake compared to other populations. It is important to distinguish what factors for acceptance and hesitancy relate to HBMs and SCTs, as these differ highly in terms of getting the vaccine for personal health or feeling obligated to vaccinate due to environmental influences. As such, further research needs to examine more details and decision-making dynamics of adolescent and youth populations demographics only.

## Study limitations

The primary limitation of this study is the inclusion of a wide age bracket for screening article purposes. Although our primary goal was to focus on youth/adolescent populations (age 15–29), we utilized a larger age bracket for screening to find more articles from database searches. However, the 21 articles used in this study include a high percentage of young population demographics found within the findings. An additional limitation is that the two screeners were tasked with filtering separate article files. Although there was conjecture and discussion on suitable articles found after screening, each screener only had to filter half of the database searches and they did not examine all articles individually within the search. Another limitation is that some countries or regions may have had access to different COVID-19 vaccinations at different timepoints, which may have affected their vaccine choice (manufacturer) and the timing of the initial vaccine compared to booster shots. A fourth limitation is that articles appearing in a non-English language were omitted from eligibility. As a result, this scoping review may have missed some important information. The final limitation is the possibility of publication bias; meaning that only published articles are based on statistically significant direction and strength.

## Conclusions

This scoping review explored the decision-making factors and reasons that adolescent and youth populations accept or refuse the vaccine. Forms of acceptance and hesitancy rely on personal characteristics, perceptions, and motivators. Although many of these decision-making dynamics relate to personal behaviours and beliefs, some instances of acceptance or hesitancy relate to government communication, public health sources, and industry vaccine information. Finally, future research needs to examine these decision-making dynamics on specific adolescent and youth population age ranges so that results are more generalizable to these specific populations. From this, we can also determine how these health behaviours are influenced by environmental or social factors, as well as personal health and susceptibility. With this research, future policies and vaccination programs may be more successful for these populations if we understand the drivers and motivators for COVID-19 vaccine status.

## Supplementary Information

**Additional file 1.** 

**Additional file 2.** 

**Additional file 3.** 

## Data Availability

All data gathered and analyzed during this study is included within this manuscript and supplementary files. Please contact Roger Blahut (rblahut@brocku.ca) for any data requests related to study.

## Abbreviations

Coronavirus Disease-2019: COVID-19
PRISMA-ScR: Preferred Reporting Items for Systematic Reviews and Meta-Analyses extension for Scoping Reviews Statement
PICOs: Participants, Interventions, Comparators, Outcomes, Study Design
HBM: Health Belief Model
SCT: Social Cognitive Theory
