# Supplementary material for: A scoping review on the decision-making dynamics for accepting or refusing the COVID-19 vaccination among adolescent and youth populations
Source: BMC Public Health. 2023 Apr 28;23:784. doi: 10.1186/s12889-023-15717-5 (PMC10141871; doi:10.1186/s12889-023-15717-5)
Supplement: Supplementary file 1 — Additional file 1. [file 12889_2023_15717_MOESM1_ESM.docx]

**Additional file 1**

**Example of database search strategy**

1 youth

2 adolescent

3 acceptance

4 refusal

5 COVID-19 vaccine

6 1 or 2 or 3 or 4 or 5

7 1 and 2 and 3 and 4 and 5
