# Supplementary material for: A scoping review on the decision-making dynamics for accepting or refusing the COVID-19 vaccination among adolescent and youth populations
Source: BMC Public Health. 2023 Apr 28;23:784. doi: 10.1186/s12889-023-15717-5 (PMC10141871; doi:10.1186/s12889-023-15717-5)
Supplement: Supplementary file 2 — Additional file 2. [file 12889_2023_15717_MOESM2_ESM.docx]

**Additional file 2**

**Supplementary table 1 -** Study attributes and characteristics

| **First author** | **Study setting** | **Recruitment** | **Study time** | **Country** | **Study population** | **Sample size** |
| --- | --- | --- | --- | --- | --- | --- |
| El-Elimat [18] | Online cross sectional, self administered questionnaire | Convenience sample, online social media | November 2020 | Jordan | General population | 3100 |
| Fojnica [23] | Cross sectional electronic survey | Emails, research and employment services, social media | January 26 to February 2 2021 | Bosnia and Herzegovina | Adult population | 10471 |
| Bendau [22] | Online cross sectional survey | Nonprobability sample, social media, news portals, website homepage | January 1 to January 11 2021 | Germany | Adult population | 1779 |
| Al-Qerem [17] | Web based cross sectional survey | Facebook all-purpose groups | October 2020 | Jordan | 18+ Jordanian population | 1144 |
| Issanov [19] | Electronic cross sectional questionnaire | Convenience sample, mass emails, social media | August to November 2020 | Kazakhstan | 18+ population | 417 |
| Elgendy [29] | Online survey | _ | April to May 2021 | Egypt | 18+ general population | 871 |
| Schwarzinger [21] | Cross sectional online survey | Stratified random sampling | June 22 to July 3 2020 | France | Representative sample working age adults 18-64 | 1942 |
| Kumari [10] | Web-based cross sectional survey | Web link sent to email or social contacts, phone interviews and Google forms for lower literacy/limited technological participants | March 13 to March 25 2021 | India | 18 years and above for representative population | 1249 |
| Wong [16] | Online self administered questionnaire | Convenience sampling, social media platforms, websites, blogs | January 4 to March 5 2021 | 17 countries world-wide | 18+ who have not been vaccinated yet | 19714 |
| Zawahrah [20] | Online cross sectional survey | Social media, university union websites | October 2020 | Palestine | 18 years and over | 1080 |
| Omar [25] | Web based cross sectional survey | Convenient sampling, social media | January 7 to March 30 2021 | Egypt | 18 years and above | 1011 |
| Brandt [12] | Open ended qualitative questions through text message | Weighted samples, MyVoice national text message poll | October 30 2020 | United States | 14 to 24 year olds | 911 |
| Paul [11] | Cross sectional design (online, face to face, in-depth interviews) | Convenience sampling, social media | January 21 to February 6 2021 | Bangladesh | 18 years and above | 1975 |
| Yasmin [26] | Cross sectional study | Social media platforms | January 28 to February 11 2021 | Pakistan | 18 + general population | 1778 |
| Boguslavsky [24] | Cross sectional online survey | Social network platforms (Vkontakte) | September 1 2021 to January 25 2022 | Russia | Aged 16 - 51 | 5822 |
| McPhedren [14] | Discrete choice experiment | Living in the UK, unvaccinated | March 25 to April 2, 2021 | United Kingdom | Aged 18 - 29 | 2012 |
| Ganczak [13] | Qualitative study - Focus group | Snowball sampling method, social media advertisements | September 1 2021 | Poland | Aged 18 - 45 - Unvaccinated at time of study | 22 |
| Baack [28] | Cross sectional study | Probability based panel surveys | March to May 2021 | United States | 18 - 39 years old | 2,726 |
| Attia [27] | Cross sectional survey, self administered questionnaire | Non-random sampling, digital channels for survey promotion, mass email, social media | December 2021 | Germany | 22- 49 years old. University students and employees | 930 (322 employees, 608 students) |
| Coulaud [15] | Cross sectional survey | Social media posts and advertisements | October 8 to December 23 2020 | France & Canada | 18 - 29 year old | 6663 (48.1% from France, remaining from Canada) |
| Burger [30] | Cross sectional analysis based on longitudinal data | Data obtained from National Income Dynamics Study: Coronavirus Rapid Mobile Survey (NIDS-CRAM), administered telephonically | February to March 2021 & April to May 2021 | South Africa | 18 and older | 5629 and 5862 (4th and 5th wave) |
