# Supplementary material for: A scoping review on the decision-making dynamics for accepting or refusing the COVID-19 vaccination among adolescent and youth populations
Source: BMC Public Health. 2023 Apr 28;23:784. doi: 10.1186/s12889-023-15717-5 (PMC10141871; doi:10.1186/s12889-023-15717-5)
Supplement: Supplementary file 3 — Additional file 3. [file 12889_2023_15717_MOESM3_ESM.docx]

**Additional file 3**

**Supplementary table 2 -** Study themes and decision-making dynamics for COVID-19 vaccine status

| **First Author** | **Factors or reasons for acceptance** [a] | **Factors or reasons for hesitancy** [b] |
| --- | --- | --- |
| El-Elimat [18] | - Younger age groups more likely to accept - Unemployed more likely to accept - Participants who took influenza vaccine this year more likely to accept COVID-19 vaccine - 59% think pharmaceutical companies will develop safe and effective COVID-19 vaccines | - Older age groups (over 35) less likely to accept - Employed individual less likely to accept - Thinking COVID-19 is conspiracy and do not trust any information were less likely to accept - Believe vaccines are unsafe - Not willing to pay - Half of participants say side effects will prevent them taking COVID-19 vaccine |
| Fojnica [23] | - Older age more likely to accept vaccine - Higher income associated with acceptance - Higher levels of education - Reasons for vaccinating: personal protection, herd immunity, avoiding travel ban, employer requirement and preserving job - Rationale for vaccine choice: efficacy proven by clinical trials (highest response) | - Younger less likely to accept vaccine - Lower income associated with hesitancy - Lower education - Reasons for refusing: covid trial regulations and appeared on market too quick (highest response), lack of trust in professional and pharmaceutical companies, vaccines harmful to overall health, religious principles, COVID-19 disease not dangerous to health |
| Bendau [22] | - Older age more likely to accept - Higher education more likely to accept - Only using official websites for information were more likely to accept vaccine - Fear of infection, health-related consequences, and COVID-19 anxiety associated with higher acceptance | - Younger age less likely to accept - Lower education less likely to accept - Not using official websites or social media reported lowest acceptance - Social and economic fears associated with unwillingness to get vaccinated |
| Al-Qerem [17] | - Higher perceived risk of COVID-19 was predictor for intending to get vaccinated - Acquaintance was infected with COVID-19, higher likelihood to get vaccinated - Those working in medical field were more likely to get vaccinated | - Concerns: vaccine efficacy, safety and side effects, vaccine rigor of testing, not convinced of effective (i.e. flu vaccine) - Additional information needed: scale of pandemic when vaccine distributed, waiting to hear more information about vaccine - Attitudes: scared to put foreign objects in body, let people more at risk take vaccine first because they don’t want to be put at risk, don’t take vaccines at all - Lack of trust: government recommends, no trust in pharmaceuticals, virus was developed by government |
| Issanov [19] | - Country of origin of vaccine: Germany was most trusted - Respondents more likely to accept vaccine that is developed outside of Kazakhstan - Above 50% of respondents believe results from evidenced-based journals - Most respondents believe in doctor’s thoughts about vaccines than family/friends - Most respondents think it’s important to follow vaccine schedule of ministry of health | - Majority of respondents with children would not vaccinate themselves or their children against COVID-19 - Country of origin of vaccine: India least trusted - If Kazakhstan developed vaccine, associated with high hesitancy - Concerns and worries about side effects and vaccine safety - If given chance to be in study trial for vaccine, 75% of respondents said “no” |
| Elgendy [29] | - Protect themselves of serious infection - Protect those close to them - Encourage friends and family to get vaccinated - Around 50% obligated to take vaccine in workplace - Those with chronic disease or knew of people hospitalized with COVID-19 infection for accepting of vaccine | - 79% of respondents think immunity after infection of virus is better than vaccine - 69% believe vaccine itself may infect them with COVID-19 - Concerned about effectiveness - Not enough clinical data - Side effects of vaccine - Majority agreed the vaccine is not for people under 18 years of age |
| Schwarzinger [21] | - Higher education status - Greater perceived severity of COVID-19 infection - More accepting of vaccine if developed in EU - Lower chance of side effect, higher acceptability - Younger and older populations more acceptable | - Lower education status - Poorer compliance with vaccines in the past - Lower perceived severity of COVID-19 infection - Country of origin for vaccine manufacturer (i.e. China was most hesitant) - Higher chance of side effect, the lower the acceptability - Middle age populations more hesitant |
| Kumari [10] | - Higher age group - Higher socio-economic status (willingness to pay for vaccine as well as accept it) - Better developed area of residence, the more likely to recommend vaccine to friends and family - Motivators include: believing the vaccine is harmless, benefits outweigh the risks, societal responsibility, many people around getting vaccinated, health professional recommendation | - Lower age group - Lower socioeconomic status - Lower developed residence - Barriers include: pharmaceutical gains, faultiness of vaccine, side effects, availability of vaccine, rapid development |
| Wong [16] | - Younger age more accepted - Higher education status - Higher effectiveness of vaccine, more acceptable - Longer threshold of effectiveness and duration | - Gradual increase with age - Lower education status - Side effects create hesitancy - Country of origin of vaccine creates hesitancy - Number of doses needed |
| Zawahrah [20] | - Perceive COVID-19 as real virus - Participants who followed safety measures were more willing to vaccinate - More willing if they thought vaccine would not cause side effects - Trust in scientists/researchers - Information should be clear and accessible | - Believe virus was fabricated or did not know what COVID-19 was - Participants who did not believe in safety measures were more hesitant - Those that thought side effects would occur |
| Omar [25] | - More preferred Pfizer vaccine, compared to Chinese and AstraZeneca - Older population more accepting | - Ministry of Health did not provide adequate information about COVID-19 vaccine - Worries about unexpected side effects - Concern on commercial profiteering - Preferred natural immunity - Mistrust of vaccine benefit - Younger population more hesitant - Perceive own health as good/very good; thus, not needing vaccine |
| Brandt [12] | - More willing to vaccinate in order to protect oneself and others (family/friends) - If experts deemed vaccination safe and effective, more willing to vaccinate - More professional sources (CDC) were most trustworthy source of information | - Safety was foremost concern - Concerns include side effects, efficacy, production rushed - Government or industry influence, conspiracies |
| Paul [11] | - Stating vaccine will protect them infection - Those interested in taking vaccine preferred Pfizer - Most respondents preferred own country vaccine (Bangladesh) - Educated more likely to vaccinate - 41 to 50 age more likely to vaccinate - Management of vaccine program: more trust in army | - Naturally protected - Side effects and temporary protection - Religious reasons - 18 to 30 less likely to vaccinate - Educated also more likely to be wary of side effects - Management of vaccine program: less trust in government capability |
| Yasmin [26] | - -More willing to take imported/international vaccine - -Suggestions to improve vaccination rates (descending rates in order): physician recommendation, further validation from studies on efficacy and safety, recommended by friends/family, government mandate, employer | - Side effects - The vaccine will not help against the virus - Follow all preventive measures seriously, so don’t need to vaccinate - Young and healthy, don’t need it |
| Boguslavsky [24] | - | - Believe in conspiracy theories - Concerned about digital innovations in healthcare - Lack of support for QR code utilization - Vexation over the opinions of citizens regarding vaccination to government officials |
| McPhedren [14] | - More willing to get vaccinated at vaccine centers or GP’s - Timing of appointments – most do not prefer after hours appointments | - Less willing to be vaccinated at a pharmacy (most) |
| Ganczak [13] | - More trust in Polish healthcare vs. Ukraine which increased vaccine acceptance - Better trained HCW’s in Poland leads to increased positive attitudes towards vaccination | - Institutional barriers for migrants relating to residence makes it difficult to receive vaccination - Difficulty finding access to vaccine information, therefore, many referred to Facebook and uncredible sources – leading to misinformation - Overall distrust in pharmaceutical companies and politicians (mostly Ukraine) - Negative influence from media showing adverse or fatal side affects from vaccine - Negative perceptions of vaccine safety, quality, efficacy and necessity - Previous experience |
| Baack [28] | - Higher education level leads to increased vaccine acceptance - Concerned about getting infected with covid lead to increased acceptance - Demographic influence | - Concern about vaccine side effects - Safety of vaccine - Believe other people need the vaccine more than they do - Lack of trust in the vaccine - Lack of information about safety, efficacy and spread - Demographic influence |
| Attia [27] | - Protection of ones own and community health increased acceptance - Easier social life lead to increased acceptance - Less covid testing increased - Employees felt compelled to vaccinate to avoid economic collapse and return to “normal” | - |
| Coulaud [15] | - High concern for family wellbeing - Higher education leads to increased vaccine acceptance - Living in metropolitan areas increased vaccine acceptance | - Individuals who felt like face coverings were unimportant to stop the spread also rejected vaccination - If individual did not know somebody who experienced severe symptoms they had higher vaccine hesitancy - Pre-existing negative attitudes towards vaccination lead to increased hesitancy (Mostly France) - Safety and efficacy - Lower education - Living in rural communities |
| Burger [30] | - Respondents who were Jewish - Those that trust in community leaders as source of COVID-19 information | - Those that reported more trust in social media - Think the vaccine is unsafe - Believe that vaccine trials were rushed - Concerns about side effects (death, blood clots, other illnesses or side effects) - Conspiracies about government or global plot |
